# Are caregiving responsibilities associated with non-attendance at breast screening?

**DOI:** 10.1186/1471-2458-10-749

**Published:** 2010-12-03

**Authors:** Heather Kinnear, Sheelah Connolly, Michael Rosato, Clare Hall, Adrian Mairs, Dermot O'Reilly

**Affiliations:** 1 Queen's University Belfast, Grosvenor Road, Belfast, Northern Ireland, UK; 2 Quality Assurance Reference Centre, Public Health Agency, 12-22 Linenhall Street, Belfast, Northern Ireland, UK

## Abstract

**Background:**

Previous research showed that deprived individuals are less likely to attend breast screening and those providing intense amounts of informal care tend to be more deprived than non-caregivers. The aim of this study was to examine the relationship between informal caregiving and uptake of breast screening and to determine if socio-economic gradients in screening attendance were explained by caregiving responsibilities.

**Methods:**

A database of breast screening histories was linked to the Northern Ireland Longitudinal Study, which links information from census, vital events and health registration datasets. The cohort included women aged 47 - 64 at the time of the census eligible for breast screening in a three-year follow-up period. Cohort attributes were recorded at the Census. Multivariate logistic regression was used to examine the relationship between informal caregiving and uptake of screening using STATA version 10.

**Results:**

37,211 women were invited for breast screening of whom 27,909 (75%) attended; 23.9% of the cohort were caregivers. Caregivers providing <20 hours of care/week were more affluent, while those providing >50 hours/week were more deprived than non-caregivers. Deprived women were significantly less likely to attend breast screening; however, this was not explained by caregiving responsibilities as caregivers were as likely as non-caregivers to attend (Odds Ratio 0.97; 95% confidence intervals 0.88, 1.06).

**Conclusions:**

While those providing the most significant amounts of care tended to be more deprived, caregiving responsibilities themselves did not explain the known socio-economic gradients in breast screening attendance. More work is required to identify why more deprived women are less likely to attend breast screening.

## Background

Breast cancer is the most common female cancer, affecting approximately 45,000 women per year in the United Kingdom (UK) [1]. Breast cancer survival rates in the UK are lower than in other parts of Europe and there is evidence to suggest that this is in part explained by the presentation at diagnosis with more advanced disease among UK patients compared to their European counterparts [2,3]. One of the most efficient ways to increase the rate of early cancer detection is through national screening programmes [4] and early diagnosis through the NHS Breast Cancer Screening Programme is considered to have contributed significantly to the overall reduction in breast cancer mortality in the UK over the past 20 years[5,6].

In the UK, the National Breast Screening Programme was set up by the Department of Health in 1988 and provides breast screening once every three years for women aged 50-64. The extension of the age range up to and including 70 took place at varying rates in different parts of the UK. England was the first, with the extension being completed by the end of 2004, with Northern Ireland being the last in 2009. Despite the service being free at the point of use and available locally, the uptake rate for Northern Ireland in 2008/09 was 73.9%. Although the effectiveness of screening programmes depends on high rates of participation, routine information on variations in uptake rates is limited. The need for more detailed information on the sources of variation in uptake of screening services has been highlighted in the final report of the Equalities Review [7] and the National Screening Programmes Information Strategy [8]. A number of studies have shown that the uptake of breast screening is associated with a variety of socio-economic factors [9,10]; for example, in a recent study, Moser et al showed for 3,185 respondents to the National Statistics Omnibus Survey, that car ownership and housing tenure were significant predictors of attendance for mammography [11]. However, it not clear why more deprived individuals are less likely to attend breast screening. One potential explanation is that the opportunity cost of accessing health services, such as breast screening, is greater for more deprived, compared to more affluent individuals. This would be the case if, for example, more deprived people had greater competing demands on their time, including providing unpaid care. Previous work has shown that those providing more intense amounts of informal care (often defined as 50 + hours/week) tend to be more deprived than non-caregivers, and also of caregivers providing fewer hours of care [12,13].

It is possible therefore that the caregiving role may reduce the amount of time available to engage in preventive health services with one study for example, noting that 80% of caregivers to people with major caregiving need were unable to leave the care recipient alone and had to organise their time around the care recipient's daily activities [14].

The evidence on caregivers' use of health services is mixed. Using data from the British Household Panel Study, one study found that females that were heavily involved in caregiving for someone within their own household had relatively less contact with a general practitioner (GP) than expected, based on non-caregivers use of GP services. In contrast, male caregivers were more likely to have contacted a GP than matched non-caregivers [15]. A study of 150 spousal caregivers of Alzheimer's disease patients and 46 matched control participants noted that caregivers experiencing more problem behaviours of their spouse had a reduced hazard for inpatient hospitalisation, which the authors hypothesised may reflect reluctance among caregivers to schedule hospital care at a time when their spouse is difficult to manage [16]. Studies too, looking at the health behaviours of caregivers have provided mixed results. In the Caregiver Healthy Effects Study, being a high-level caregiver (defined as having a spouse with an activity of daily living impairment) was found to significantly increase the likelihood of not getting enough rest, not having enough time to exercise, not having enough time to recuperate from illness, and forgetting to take prescription medications, compared to non-caregivers [17].

Alternatively, a study of 272 caregivers and 917 non-caregivers who were members of the Kaiser Foundation Health Plan in Northern California found that caregivers were more likely than non-caregivers to engage in preventative health behaviours [18], while a recent study in Hawaii, Kansas and Washington examining modifiable health behaviours found no difference between caregivers and non-caregivers [19]. The aims of this study were twofold: To determine if caregiving responsibilities were associated with reduced attendance at breast screening and to determine if socio-economic gradients in breast screening attendance were explained by caregiving responsibilities.

## Methods

Data for breast screening invitations and attendance were extracted from the central organising authority (Northern Ireland Breast Screening Quality Assurance Reference Centre) and linked to a census-based longitudinal study (the Northern Ireland Longitudinal Study - NILS) using a unique health care identifier, common to both datasets. The combined research dataset was entirely anonymous and held in a safe setting within the Northern Ireland Statistics and Research Agency. A one-way encryption algorithm was applied to the unique health care identifier on both datasets. Ethical approval for the project was granted from the Office for Research Ethics Committees Northern Ireland.

The NILS is a large-scale data linkage study which has been created by linking individual level administrative and statistical data. Information is linked over time on people from Census, vital events (births, deaths and marriages) and GP health registration datasets. The NILS consists of an approximate 28% sample of the Northern Ireland population (approximately 450,000 individuals), with sample members chosen on having one of 104 birth dates. The cohort for this analysis included all women aged between 47 and 64 (as of the Census date in 2001 - 29^th ^April) eligible for breast screening between 29^th ^April 2001 and 28^th ^April 2004. To allow for delays in attending for screening, a six month period was added, extending the effective period of analysis to the 28^th ^October 2004.

The caregiving, demographic, socio-economic and health characteristics were as described on the census record. The extent of caregiving was determined from the census question: "*Do you look after, or give any help or support to family members, friends, neighbours or others because of: long term physical or mental ill-health or disability; problems related to old age*?" with respondents choosing from: no caregiving responsibilities; 1-19 hours/week; 20-49 hours/week; or 50 or more hours/week.

A number of characteristics known to be associated with uptake of breast screening [20] were included in the analysis. Demographic characteristics included age (47-54; 55-64) and marital status (single; married; separated/widowed/divorced). Socio-economic status was assessed using housing tenure (categorised as owner-occupier and renter), household car availability (grouped as two or more, one or none), and economic activity [21] (categorised as employed and currently unemployed). These were then combined into a five point composite indicator of socio-economic status, with 1 representing the most advantaged (corresponding to an owner-occupier, with access to two or more cars and employed in a professional/managerial job) and 5 representing the most disadvantaged (corresponding to those living in rented accommodation, with no car access and currently unemployed). A question on general health (GH) in the year preceding the census (offering three potential responses - good, fairly good and not good) was asked in the 2001 census, and included in the analysis. As previous work has shown that breast screening uptake tends to be lower in larger UK cities [22,23], an indicator of area of residence - distinguishing Belfast Metropolitan Area (BMA) (approximate population 650,000) from the rest of Northern Ireland - was also included.

All analyses were carried out using STATA version 10. The analysis is presented in three stages: a description of the demographic and socioeconomic characteristics of women invited for screening by caregiving status; an analysis of the percentage uptake of screening for various demographic, socio-economic and caregiving groups; and a multivariate unconditional logistic regression analysis of the variation in uptake of screening according to the level of deprivation and the amount of care provided. Adjustment was made for all known confounders that have previously been shown to predict screening attendance.

## Results

37,211 women were invited for breast screening during the study period, of whom 27,909 (75%) attended. Of those attending, 24.6% reported being caregivers at the time of the 2001 Census, while 21.9% of those not attending breast screening, reported being caregivers.

Table 1 shows the demographic, socio-economic and health characteristics of the study population, grouped by caregiving status. A higher proportion of caregivers (regardless of the intensity of caregiving) were married, compared with non-caregivers although there were more single women in the group of caregivers who provided more than 50 hours/week compared to non-caregivers and those caregivers proving between 1-49 hours/week. The pattern was mixed in terms of deprivation with more caregivers providing 50 + hours/week falling in the three most deprived categories (30.5%) compared to caregivers providing between 1-19 hours/week (11.3%), those providing between 20-49 hours/week (20.4%) and non-caregivers (27.0%). In keeping with the known association between caregiving and socio-economic status [13,14], caregivers providing the fewest hours of care reported better health than non-caregivers and those providing a more intensive amount of care: 91% of those providing between 1 and 19 hours of care reported their health as good or fairly good compared to 77% of non-caregivers, 87% of those providing between 20 and 49 hours of care and 84% of those providing 50 or more hours of care per week.

**Table 1:** Characteristics of those invited for screening (n = 37,211), by caregiving status

	*Non-caregiver (n = 28,308) 76.1%*	*1-19 hrs/week (n = 5,013) 13.5%*	*20-49 hrs/week (n = 1,389) 3.7%*	*50 + hrs/week (n = 2,501) 6.7%*
**Age**				
47-54	12,714 (44.9%)	2,661 (53.1%)	690 (49.7%)	1,144 (45.7%)
55-64	15,594 (55.1%)	2,352 (46.9%)	699 (50.3%)	1,357 (54.3%)
**Marital status**				
Single	2,191 (7.7%)	311 (6.2%)	122 (8.8%)	261 (10.4%)
Married	19,959 (70.5%)	3,982 (79.4%)	1,076 (77.5%)	1,963 (78.5%)
Separated/Widowed/Divorced	6,158 (21.8%)	720 (14.4%)	191 (13.7%)	277 (11.1%)
**Deprivation**				
1 Least deprived	10,780 (38.1%)	2,777 (55.4%)	560 (40.3%)	744 (29.7%)
2	9,885 (34.9%)	1,668 (33.3%)	545 (39.2%)	995 (39.8%)
3	4,142 (14.6%)	367 (7.3%)	171 (12.3%)	498 (19.9%)
4	2,902 (10.3%)	173 (3.5%)	100 (7.2%)	226 (9.0%)
5 Most deprived	599 (2.1%)	28 (0.6%)	13 (0.9%)	38 (1.5%)
**General health**				
Good	14,223 (50.2%)	3,101 (61.9%)	702 (50.5%)	1,131 (45.2%)
Fairly good	7,700 (27.2%)	1,509 (30.1%)	504 (36.3%)	960 (38.4%)
Not good	6,385 (22.6%)	403 (8.0%)	183 (13.2%)	410 (16.4%)
**Settlement type**				
Rest of Northern Ireland	18,691 (66.0%)	3,074 (61.3%)	856 (61.6%)	1,657 (66.3%)
Belfast Metropolitan Area (BMA)	9,617 (34.0%)	1,939 (38.7%)	533 (38.4%)	844 (33.8%)

Table 2 shows the odds of attending screening for various demographic, socio-economic and health characteristics, including caregiving status. Those aged 55 to 64 were less likely to attend breast screening than those aged 47 to 54, although this was no longer significant when adjustment was made for the demographic, socio-economic, health characteristics and area factors of the cohort. Women who were single or separated, widowed or divorced were significantly less likely to attend for screening than married women. There was a strong dose response relationship between socio-economic status and screening attendance with those in the most deprived group about 66% less likely to attend than their more affluent peers (OR 0.33; 95% CI 0.28-0.39). Having adjusted for the demographic, socio-economic and baseline health characteristics of the cohort, women residing within the BMA were almost 40% less likely to attend screening than women living in the rest of Northern Ireland.

**Table 2:** Variation in breast screening attendance

	*Number in cohort (% screening uptake)*	*Adjusted for age only OR (95% CI)*	***Fully Adjusted Model1 ***^**a**^***OR(95%CI)***	***Fully adjusted Model2 ***^**b**^***OR(95%CI)***
**Age**				
47-54	17,209 (76.0)	1.00	1.00	1.00
55-64	20,002 (74.1)	**0.91 (0.87-0.96)**	0.96 (0.91-1.01)	0.95 (0.91-1.01)
**Marital status**				
Married	26,980 (78.0)	1.00	1.00	1.00
Single	2,885 (66.6)	**0.56 (0.52-0.61)**	**0.75 (0.69-0.82)**	**0.75 (0.69-0.82)**
Separated//Widowed/Divorced	7,346 (67.4)	**0.59 (0.55-0.62)**	**0.84 (0.79-0.90)**	**0.84 (0.79-0.89)**
**Deprivation**				
1 Least deprived	14,861 (80.3)	1.00	1.00	1.00
2	13,093 (74.9)	**0.78 (0.74-0.83)**	**0.86 (0.81-0.91)**	**0.87 (0.81-0.92)**
3	5,178 (60.0)	**0.51 (0.47-0.54)**	**0.60 (0.55-0.65)**	**0.61 (0.56-0.65)**
4	3,401 (78.0)	**0.32 (0.30-0.35)**	**0.41 (0.38-0.45)**	**0.42 (0.38-0.46)**
5 Most deprived	678 (62.4)	**0.25 (0.22-0.30)**	**0.33 (0.28-0.38)**	**0.33 (0.28-0.39)**
**General health**				
Good	19,157 (77.6)	1.00	1.00	1.00
Fairly good	10,673 (75.2)	0.88 (0.84-0.93)	0.99 (0.94-1.05)	0.99 (0.94-1.05)
Not good	7,381 (68.0)	**0.62 (0.58-0.66)**	**0.82 (0.77-0.87)**	**0.83 (0.77-0.88)**
**Settlement type**				
Rest of Northern Ireland	24,278 (78.6)	1.00	1.00	1.00
Belfast Metropolitan Area (BMA)	12,933 (68.2)	**0.59 (0.56-0.63)**	**0.63 (0.60-0.66)**	**0.63 (0.60-0.66)**
**Caregiving status**				
Non-caregiver	28,308 (74.4)	1.00		1.00
1-19 hours/week	5,013 (79.0)	**1.28 (1.19-1.38)**		**1.11 (1.03-1.20)**
20-49 hours/week	1,389 (76.0)	1.09 (0.96-1.24)		1.03 (0.91-1.17)
50 + hours/week	2,501 (73.9)	0.98 (0.89-1.07)		0.97 (0.88-1.06)

^a ^Adjusted for age, marital status, deprivation, baseline health and settlement type, but not caregiving status

^b ^Adjusted for age, marital status, deprivation, baseline health, settlement type and caregiving status

In general, caregiving was not associated with screening attendance, with the exception of those caregivers providing less than 20 hours unpaid care/week, who were more likely than non-caregivers to attend, though this was reduced with further adjustment for socio-economic status (OR 1.11; 95% CI 1.03-1.20). Caregivers providing 50 or more hours/week were as likely as non-caregivers to attend breast screening (OR 0.97; 95% CI 0.88-1.06). Adjusting for caregiver status did not attenuate the socio-economic gradient in attendance.

## Discussion

This large-scale population-based study confirmed the strong dose response relationship between socio-economic status and breast screening attendance, with the most deprived significantly less likely to attend than their more affluent peers. It also showed that caregiving was not associated with screening attendance, with the exception of those caregivers providing less than 20 hours unpaid care/week, who were more likely than non-caregivers to attend. While there were more caregivers providing 50 + hours/week falling in the three most deprived groups compared to both non-caregivers and caregivers providing between 1-49 hours/week, they were as likely as non-caregivers to attend breast screening and adjusting for caregiver status did not attenuate the socio-economic gradient in attendance.

These results are in keeping with a number of other studies which have found that caregivers had similar or higher use of preventive health services than non-caregivers. Scharlach et al, for example, found that caregivers were more likely than non-caregivers to eat breakfast daily, get flu shots and receive pneumonia vaccines, while no significant differences were found among caregivers and non-caregivers for a number of health practices including smoking and receiving a mammogram [18]. McGuire and colleagues found that caregivers and non-caregivers did not differ with regard to fruit and vegetable consumption, smoking status or alcohol consumption, but that caregivers were more likely to adhere to physical activity recommendations than non-caregivers [19]. In addition, this study has confirmed previous findings of significant variations in uptake of breast screening by age and marital [20], and socio-economic status [11].

These findings are in contrast, however to other studies which have suggested that caregivers have less time available to look after their own health. Shaw et al for example, found that caregivers dealing with problem behaviours were less likely to be hospitalised than those dealing with fewer problem behaviours [16], while Burton and colleagues found a significant association between caregiving level and inadequate exercise, inadequate rest and forgetting to take medication [17]. In both of these studies, caregivers were grouped by the needs or behaviours of the care recipient rather than the amount of time spent caregiving, and it likely that these studies are identifying the most stressed caregivers, for whom the caregiving role may limit their ability to look after their own health. While it may be expected that the time commitment associated with a caregiving role would act as a deterrent to accessing preventive health services such as breast screening, there are a number of reasons why this may not be the case. Alternatively, it is also possible that caregivers may look after their health more than they would otherwise as they know that the care recipient is dependent on them to remain healthy in order to remain in the caregiving role [18]. Other studies have demonstrated that those providing less than 20 hours of caregiving per week differ from those who provide more intensive levels of caregiving; for example they are more affluent and more likely to own a car and it is likely that their higher screening attendance is related to these differences. Caregivers may be more likely to access health services as their caregiving role may bring them more in contact with the health services, either to seek help associated with their caregiving responsibilities or to seek advice about the care recipient's health [15,24].

"It is known that those from deprived backgrounds are less likely to use screening services [11] but it is not known exactly why this is the case. Car access and housing tenure have been shown to be predictive of attendance at screening but why this is so is not known [11]. No other research before has looked at whether those in deprived backgrounds are more likely to be caregivers. This analysis clearly demonstrates that this is not the case. Further research is needed to further understand why social gradients in attendance occur. This analysis has a number of strengths including its large, representative study population, accurate information on breast screening uptake and information on a wide range of covariates; however, the limitations of the analysis need to be highlighted. Caregiving status was only measured at one point in time and it is possible that during the period of study some will have ceased while others will have entered the caregiving role [25,26]. However as the maximum period for an invitation to attend screening was three years this is not likely to be a significant problem. The amount of information related to caregiving in the census was limited; there was no indication on the type of care provided, or the degree of illness or disability of the care recipient or the stress associated with the caregiving role. However, it is likely that hours of care provided is a good proxy for the demands of the caregiving role especially for those women providing the most intense amounts of care (50 and over hours per week). Finally, this analysis is limited only to female caregivers and the results may not be generalisable to male or older caregivers or to other health protection activities.

## Conclusions

This analysis has shown that caregivers are as likely, if not more likely, to access breast screening than non-caregivers. However, other research has demonstrated that some caregivers who are experiencing significant levels of stress have poorer health outcomes and further work is needed to specifically examine their use of health services [27].

## Competing interests

The authors declare that they have no competing interests.

## Authors' contributions

HK and DOR designed the study. HK with the assistance of MR and SC undertook all of the analysis. All authors contributed to the interpretation of the results and the writing of the paper. All authors had full access to all of the data during the course of the study and can be held responsible for the integrity and accuracy of the data. All authors reads and approved the final manuscript.

## Authors' information

HK - PhD Research Fellow in the School of Medicine, Dentistry and Biomedical Sciences, Queen's University Belfast, Northern Ireland, UK

SC - PhD Research Fellow in the School of Medicine, Dentistry and Biomedical Sciences, Queen's University Belfast, Northern Ireland, UK

MR - MSc Research Fellow in the School of Medicine, Dentistry and Biomedical Sciences, Queen's University Belfast, Northern Ireland, UK

CH - Information Officer, Public Health Agency, Belfast, Northern Ireland, UK

AM - MD Director of the Quality Assurance Reference Centre, Public Health Agency, Belfast, Northern Ireland, UK

DOR - MD Senior Lecturer in the School of Medicine, Dentistry and Biomedical Sciences, Queen's University Belfast, Northern Ireland, UK

## Pre-publication history

The pre-publication history for this paper can be accessed here:

http://www.biomedcentral.com/1471-2458/10/749/prepub
